# Characterization of Age-Related Differences in the Human Choroid Plexus Volume, Microstructural Integrity, and Blood Perfusion Using Multiparameter Magnetic Resonance Imaging

**DOI:** 10.3389/fnagi.2021.734992

**Published:** 2021-09-17

**Authors:** Joseph S. R. Alisch, Matthew Kiely, Curtis Triebswetter, Maryam H. Alsameen, Zhaoyuan Gong, Nikkita Khattar, Josephine M. Egan, Mustapha Bouhrara

**Affiliations:** Laboratory of Clinical Investigation, National Institute on Aging, National Institutes of Health, Baltimore, MD, United States

**Keywords:** choroid plexus, aging, magnetic resonance imaging, relaxometry mapping, diffusion tensor imaging (DTI)

## Abstract

The choroid plexus (CP) is an important cerebral structure involved in cerebrospinal fluid production and transport of solutes into the brain. Recent studies have uncovered the involvement of the CP in neurological disorders such as Alzheimer’s disease and multiple sclerosis. However, our understanding of human age-related microstructural and functional changes in the CP with aging and neuropathology is limited. In this cross-sectional study, we investigated age and sex differences in the CP structure and function using advanced quantitative magnetic resonance imaging methodology in a large cohort (*n* = 155) of cognitively unimpaired individuals over a wide age range between 21 and 94 years. Our analysis included volumetric measurements, relaxometry measures (*T*_1_ and *T*_2_), diffusion tensor imaging (DTI) measures of fractional anisotropy (FA) and mean diffusivity (MD), as well as measures of cerebral blood flow (CBF). Our results revealed that CP volume was increasing with advancing age. We conjecture that this novel observation is likely attributed to alterations in the CP microstructure or function as well as to ventriculomegaly. Indeed, we also found that CBF was lower with advanced age, while, consistent with previous studies, *T*_1_, *T*_2_ and MD were higher, and FA was lower with advanced age. We attribute these functional and microstructural differences to a deteriorated CP structural integrity with aging. Furthermore, our relaxometry and DTI measures were found to be associated with differences in blood perfusion revealing lower microstructural integrity with lower CBF. Finally, in agreement with literature, sex-related differences in MD and CBF were statistically significant. This work lays the foundation for ongoing investigation of the involvement of CP in neurodegeneration.

## Introduction

The choroid plexus (CP) modulates brain homeostasis through several processes including cerebral spinal fluid (CSF) secretion and expression of various proteins (Lun et al., 2015; Hofman and Chen, 2016). Specifically, the CP secretes numerous neurotrophic factors implicated in neurogenesis, vascularity regulation, maintenance of brain function, and signaling molecules including neuroinflammatory cytokines (Arnaud and Di Nardo, 2016; Mazucanti et al., 2019). The ubiquitous multifunctional role of the CP was highlighted in a recent study by Mazucanti et al. (2019), indicating that insulin is produced in the CP and modulated by serotonin. Structurally, the CP is a monolayer of epithelial cells connected by tight junctions situated within the ventricles that regulate the passage of solutes, proteins, and inflammatory cells to and from the CSF (Lun et al., 2015). Fenestrated capillaries are separated from the epithelial cells by stroma, and allow filtration of fluids, thereby forming the blood-CSF-barrier (BCSFB) (Liddelow, 2015).

According primarily to postmortem and animal studies, during normal aging, the CP undergoes several alterations, including morphological changes to the epithelial cells (Shuangshoti and Netsky, 1970; Serot et al., 1997, 2001; Marques et al., 2013), accumulation of calcifications (Yalcin et al., 2016), increased T helper type 2 (Th2) response (Baruch et al., 2013), dysfunction in CSF production and clearance (May et al., 1990; Chiu et al., 2012), and iron deposition (Joseph-Mathurin et al., 2013). Moreover, the CP is highly vascularized and contains energy-demanding epithelial cells, rendering it vulnerable to hypoxia, ischemia and hypoperfusion, potentially leading to cell death, increased permeability and dysfunction of the BCSFB (Chen et al., 2009), and reduced secretion of trophic factors and CSF generation. These changes have been associated with neurodegenerative diseases including Alzheimer’s disease (AD) (Kadel et al., 1990; Cornford et al., 1997; Ennis and Keep, 2006; Krzyzanowska and Carro, 2012; Marques et al., 2013; Kaur et al., 2016; Hubert et al., 2019). The CP is a small region and difficult to distinguish from the surrounding tissue, requiring high resolution imaging and careful postprocessing manipulation including CP volume segmentation; therefore, despite its mediation of several critical processes, the effect of normal aging on CP microstructure and perfusion has not been well studied *in-vivo* in humans.

Previous magnetic resonance imaging (MRI)-based studies have demonstrated that subjects with psychiatric disorders (Lizano et al., 2019), complex regional pain syndrome (Zhou et al., 2015), multiple sclerosis (Ricigliano et al., 2021) and stroke (Egorova et al., 2019), have larger CP volumes compared to healthy controls, whereas studies evaluating CP size in participants without any sign of pathology have only looked at thickness. Indeed, although Madhukar et al. (2012) and İmamoğlu et al. (2013) showed that CP thickness was consistent in pediatric and adult cohorts, changes in CP volume in normal aging has not been characterized. Moreover, to the best of our knowledge, MRI-based microstructural and perfusion differences in the CP with age or sex have only been documented by two studies. In this pioneering work, Alicioglu et al. (2017) assessed the mean diffusivity (MD), measured using diffusion tensor imaging (DTI) MRI, in human CP across various age ranges and found that the eldest participants exhibit significantly higher MD than the younger participants; this result was interpreted as an increase in diffusion in CP with age indicating a loss of structural integrity resulting in increased leakiness of the epithelial cell junctions. In contrast, Bouzerar et al. (2013) reported a decrease in capillary permeability with age. Additionally, the authors observed no significant difference in CP CBF with age using dynamic susceptibility contrast enhanced MRI (Bouzerar et al., 2013). The discrepancy in results may be due to cerebral lesions present in patient data or the limited age range or size of the cohorts studied.

Several quantitative MRI techniques have been used to evaluate cerebral tissue microstructure *in-vivo* with aging and neuropathology (Grieve et al., 2007; Hasan et al., 2010; Alisch et al., 2021). Longitudinal and transverse relaxation times (*T*_1_ and *T*_2_) depend on water mobility as well as macromolecular tissue composition including iron content; therefore, changes in *T*_1_ or *T*_2_ are directly associated with cerebral microstructural tissue changes (Deoni, 2010). Further, fractional anisotropy (FA) and MD, derived from DTI, can be used to probe cerebral tissue microstructural integrity based on water content and mobility (Alexander et al., 2007; Grech-Sollars et al., 2015). Finally, cerebral blood flow (CBF) in the CP can be reliably determined using arterial-spin labeling (ASL) MR imaging (Evans et al., 2020; Zhao et al., 2020). In the current study, we investigated volumetric differences with age as well as microstructural and perfusion characteristics of the CP in a large cohort of well-characterized cognitively unimpaired adults (*n* = 155) across the extended age range of 21–94 years. Our main goals were to investigate the effect of age and sex on relaxation times, DTI indices and CBF, to investigate the effect of age and sex on CP microstructure and function, and to develop further insights into CP maturation and degeneration with aging.

## Materials and Methods

### Participants

The MRI protocol was approved by the MedStar Research Institute and the National Institutes of Health Intramural Ethics Committees, and all examinations were performed in compliance with the standards established by the National Institutes of Health Institutional Review Board. Participants were drawn from the Baltimore Longitudinal Study of Aging (BLSA) (Shock, 1984; Ferrucci, 2008), and the Genetic and Epigenetic Signatures of Translational Aging Laboratory Testing (GESTALT) study. The study populations, experimental design, and measurement protocols of the BLSA have been previously reported (Shock, 1984; Ferrucci, 2008). The BLSA is a longitudinal cohort study funded and conducted by the NIA Intramural Research Program (IRP). Established in 1958, the BLSA enrolls community-dwelling adults with no major chronic conditions or functional impairments. The GESTALT study is also a study of healthy volunteers, initiated in 2015, and funded and conducted by the NIA IRP. The goal of the BLSA and GESTALT studies is to evaluate multiple biomarkers related to aging. We note that the inclusion and exclusion criteria for these two studies are essentially identical. Participants underwent testing at the NIA’s clinical research unit and were excluded if they had metallic implants, neurologic or medical disorders. Further, all participants underwent a battery of cognitive tests and participants with cognitive impairment were excluded (O’Brien et al., 2009). Clinical and neuropsychological data from participants were reviewed at a consensus conference if they screened positive on the Blessed Information Memory Concentration (BIMC) score (Fuld, 1978) (score ≥4), if their Clinical Dementia Rating (CDR) (Morris, 1993) score was ≥0.5 using subject or informant report, or if concerns were raised about their cognitive status. The CDR was administered to PET neuroimaging study and autopsy study participants at each visit and to the remaining participants if they scored 4 or more BIMC errors. In addition, all autopsy study participants were evaluated by case conference upon death or withdrawal. Diagnoses of dementia and AD were based on DSM-III-R (American Psychiatric Association, 1994) and the National Institute of Neurological and Communication Disorders and Stroke — AD and Related Disorders Association (McKhann et al., 1984) criteria, respectively. Mild cognitive impairment (MCI) was based on the Petersen criteria (Petersen et al., 1999) and diagnosed when (1) cognitive impairment was evident for a single domain (typically memory) or (2) cognitive impairment in multiple domains occurred without significant functional loss in activities of daily living. The final cohort consisted of 155 cognitively unimpaired volunteers ranging in age from 21 to 94 years (53.3 ± 21.3 years) of which 90 were men (54.9 ± 22.2 years) and 65 were women (52.3 ± 20.0 years), after exclusion of seven participants with cognitive impairment. Age (*p* > 0.1) did not differ significantly between men and women. The number of participants per age-decade was 23 (11 females) within 20–29 years, 22 (7 females) within 30–39 years, 40 (21 females) within 40–49 years, 12 (3 females) within 50–59 years, 10 (6 females) within 60–69 years, 19 (7 females) within 70–79 years, 25 (10 females) within 80–89 years, and 4 (0 females) within 90–99 years. Experimental procedures were performed in compliance with our local Institutional Review Board, and participants provided written informed consent.

### Data Acquisition

Magnetic resonance imaging scans were performed on a 3T whole body Philips MRI system (Achieva, Best, Netherlands) using the internal quadrature body coil for transmission and an eight-channel phased-array head coil for reception. For each participant, the imaging protocol for longitudinal and transverse relaxation times (T1 and T2), DTI metrics, and CBF imaging was as follow:

*T*_1_ and *T*_2_ Mapping (Bouhrara and Spencer, 2015, 2016, 2017; Bouhrara et al., 2016): 3D spoiled gradient recalled echo (SPGR) images were acquired with flip angles of (2, 4, 6, 8, 10, 12, 14, 16, 18, and 20°), echo time (TE) of 1.37 ms, repetition time (TR) of 5 ms, and acquisition time of ∼5 min, as well as 3D balanced steady state free precession (bSSFP) images acquired with flip angles of (2, 4, 7, 11, 16, 24, 32, 40, 50, and 60°), TE of 2.8 ms, TR of 5.8 ms, and acquisition time of ∼6 min. The bSSFP images were acquired with radiofrequency excitation pulse phase increments of 0 or π in order to account for off-resonance effects (Deoni, 2011). All SPGR and bSSFP images were acquired with an acquisition matrix of 150 × 130 × 94, voxel size of 1.6 mm × 1.6 mm × 1.6 mm. Further, we used the double-angle method (DAM) to correct for excitation radio frequency inhomogeneity (Stollberger and Wach, 1996). For this, two fast spin-echo images were acquired with flip angles of 45 and 90°, TE of 102 ms, TR of 3,000 ms, acquisition voxel size of 2.6 mm × 2.6 mm × 4 mm, and acquisition time of ∼4 min. All images were acquired with field of view (FoV) of 240 mm × 208 mm × 150 mm. The total acquisition time was ∼21 min.

Fractional anisotropy (FA) and mean diffusivity (MD) maps were derived from the DTI dataset. DTI protocol consisted of diffusion-weighted images (DWI) acquired with single-shot EPI, TR of 10,000 ms, TE of 70 ms, two b-values of 0 and 700 s/mm^2^, with the latter encoded in 32 directions, acquisition matrix of 120 × 104 × 75, and acquisition voxel size of 2 mm × 2 mm × 2 mm. Two images at *b* = 0 s/mm^2^ were acquired. Images were acquired with FoV of 240 mm × 208 mm × 150 mm.

Pseudo continuous arterial spin labeling (pCASL) for CBF Mapping (Alsop et al., 2015): 2D control, labeled, and proton density (PD) images were acquired with incorporation of background suppression and single shot-EPI with FoV of 220 mm × 210 mm × 120 mm, spatial resolution of 2.5 mm × 2.5 mm × 5 mm, TE of 15 ms, TR of 7.5 s, labeling duration of 1.8 s, post-labeling delay of 2 s, 30 signal averages, and readout duration of 21.2 ms. The total acquisition time was ∼12 min.

All images were reconstructed to a voxel size of 1 mm × 1 mm × 1 mm. We emphasize that all MRI studies and ancillary measurements were performed with the same MRI system, running the same pulse sequences, at the same facility, and directed by the same investigators for both BLSA and GESTALT participants.

### Data Processing

For each participant, the scalp and other non-parenchymal regions within the images were eliminated using the FMRIB Software Library (FSL) using an input image consisting of the SPGR images averaged over all 10 flip angles (Jenkinson et al., 2012); this provides high tissue contrast and signal-to-noise ratio for accurate segmentation.

#### Choroid Plexus and Lateral Ventricle Volumes Calculation

**FIGURE 1:**
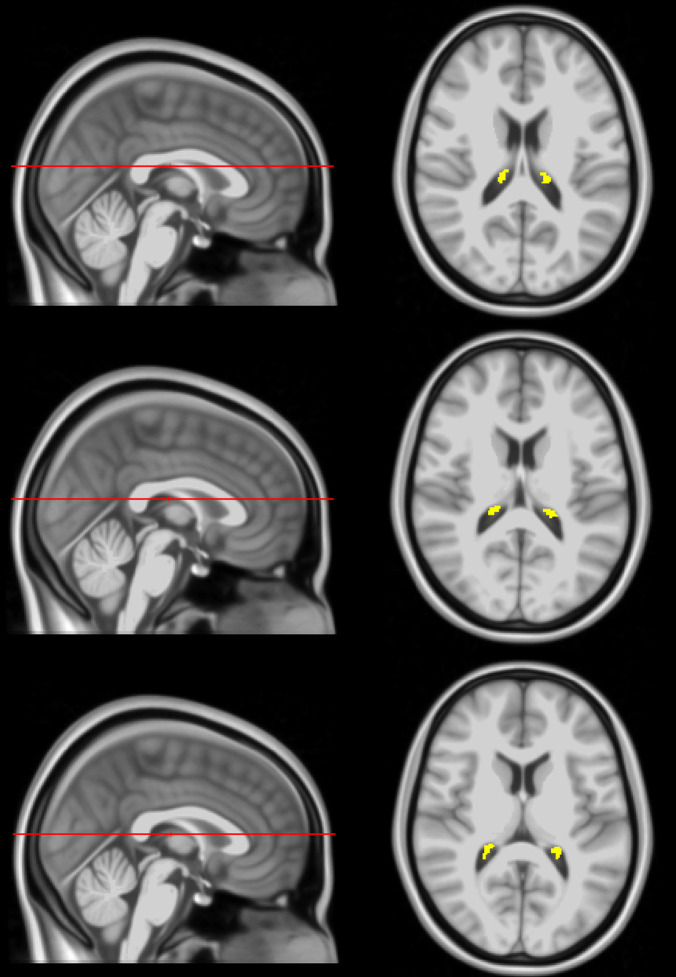
Example of CP ROI location, extracted using FreeSurfer, in the posterior portion of the lateral ventricles projected on the MNI atlas at three different slices. The right side shows an axial view of the segmentation while the left side shows the sagittal view of the slice location.

For each participant, corresponding *T*_1_-weighted SPGR images were used. Specifically, the FreeSurfer Aseg Atlas (Fischl et al., 2002) was non-linearly registered to the SPGR image averaged over all flip angles using the cortical reconstruction (*recon-all*) pipeline from the Freesurfer v7.1.1 software^1^ (Fischl, 2012). Volumetric measurements were then extracted from the CP and lateral ventricles (LV) regions of interest (ROIs) (Figure 1). This method has been used in several other studies indicating reliable CP and LV segmentations (Grech-Sollars et al., 2015; Zhou et al., 2015; Chaddad et al., 2017; Egorova et al., 2019; Lizano et al., 2019; Tadayon et al., 2020). All CP and LV ROIs were thoroughly examined and corrected manually when needed.

#### Relaxation Times Mapping

Using the FSL software (Jenkinson et al., 2012), all SPGR, bSSFP, and DAM images were linearly registered to the SPGR image acquired at a flip angle of 8^*o*^, and the derived transformation matrix was then applied to the original SPGR, bSSFP, and DAM images. Next, a whole-brain T_1_ map was generated from the co-registered SPGR dataset using the DESPOT1 analysis and assuming a single relaxing component using the stochastic regions contraction (SRC) algorithm while correcting for transmit field, B_1_, inhomogeneities (Berger and Silverman, 1991; Deoni et al., 2003). The B_1_ map was generated from the co-registered fast spin-echo using the DAM approach (Stollberger and Wach, 1996). Further, using these derived T_1_ and B_1_ maps as input parameters, a whole-brain T_2_ map was generated from the co-registered bSSFP dataset using the DESPOT2 analysis and assuming a single component using the SRC algorithm (Berger and Silverman, 1991; Deoni et al., 2003). B_1_, T_1_, and T_2_ maps were generated using in-house MATLAB scripts. All these MATLAB codes are available upon request. Next, using FreeSurfer, the SPGR image averaged over all flip angles for each participant was registered using non-linear registration to FreeSurfer’s Aseg atlas and the derived transformation matrix was then applied to the corresponding T_1_ and T_2_ maps. Finally, the mean T_1_ and T_2_ values in the CP ROI were calculated.

#### Fractional Anisotropy and Mean Diffusivity Mapping

The DW images were corrected for eddy current and motion effects using affine registration as implemented in FSL (Jenkinson et al., 2012), and then registered to the DW image obtained with *b* = 0 s/mm^2^. Moreover, whole-brain FA and MD maps were derived from the co-registered DWI data. Here we used the *DTIfit* tool implemented in FSL to calculate the eigenvalue maps which were used to calculate FA and MD (Basser and Jones, 2002). Then, for each participant, the DW image obtained at *b* = 0 s/mm^2^ was non-linearly registered to FreeSurfer’s Aseg atlas and the calculated matrix of transformation was applied to the corresponding FA and MD maps. Finally, the mean FA and MD values in the CP ROI were calculated.

#### Cerebral Blood Flow Mapping

For each participant, a whole-brain CBF map was generated from the corresponding pCASL dataset (Alsop et al., 2015). The PD image was non-linearly registered to FreeSurfer’s Aseg atlas and the computed transformation matrix was then applied to the corresponding CBF map. Finally, the mean CBF value in the CP ROI was calculated.

### Statistical Analysis

The effect of age and sex on *T*_1_, *T*_2_, FA, MD, CBF, LV, and CP volume was investigated using multiple linear regression with the mean *T*_1_, *T*_2_, FA, MD, CBF, LV, or CP volume value as the dependent variable and sex, age, and age^2^ as the independent variables, after mean centering of age. Additionally, the effect of CBF on *T*_1_, *T*_2_, FA, or MD was also evaluated using multiple linear regression while accounting for age as a covariate. The threshold for statistical significance was *p* < 0.05. All calculations were performed with MATLAB (MathWorks, Natick, MA, United States).

## Results

**FIGURE 2:**
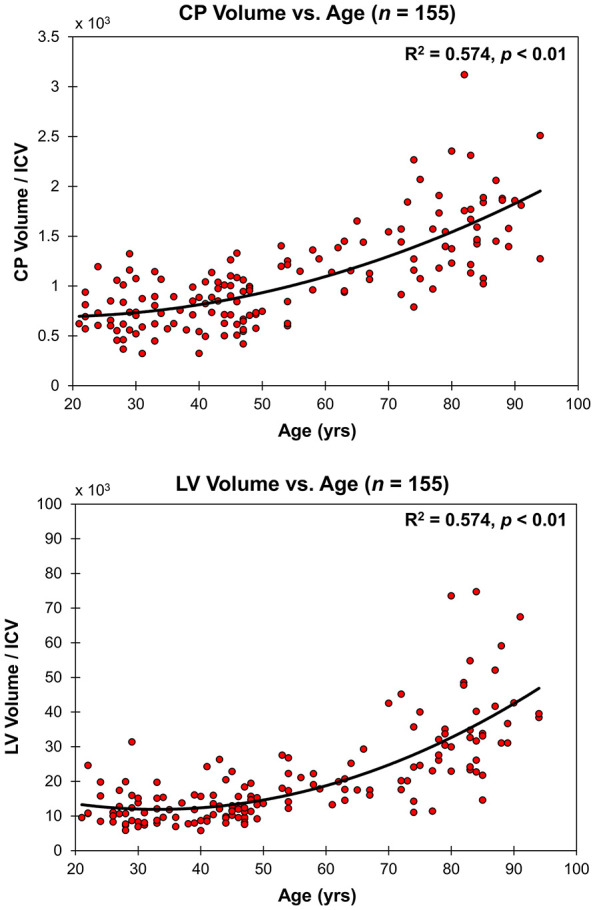
Regressions of CP and LV volumes, after correcting for total intracranial volume (ICV), with age. For each plot, the coefficient of determination, *R*^2^, and *p*-value, *p*, are reported. LV and CP volume showed non-linear increases with age.

Figure 2 shows the relationship between the CP volume or the LV volume, after correcting for the total intracranial volume, and age for all participants (*n* = 155). The CP and LV volumes showed significant quadratic effects of age (Table 1), age^2^, with both plots showing a non-linear increase in CP or LV volume with age (Figure 2 and Table 1). The effect of sex on the CP and LV volumes was statistically non-significant (Table 1).

**TABLE 1:** Coefficients of regression ± standard error (SE) and *p*-values of age, age^2^ and sex incorporated in the multiple linear regression analysis for each MR measure.

	Multiple linear regression model parameters					
	*β*_*age*_ ± SE_*age*_	*p*_*age*_	*β*_*age*_^2^ ± SE_*age*_^2^	*p*_*age*_^2^	*β*_*sex*_ ± SE_*sex*_	*p*_*sex*_
CP volume	1.56 × 10^–5^ ± 1.27 × 10^–6^	**<0.01**	1.96 × 10^–7^ ± 6.96 × 10^–8^	**<0.01**	6.95 × 10^–5^ ± 5.19 × 10^–5^	>0.1
LV volume	3.90 × 10^–4^ ± 3.48 × 10^–5^	**<0.01**	9.31 × 10^–6^ ± 1.91 × 10^–6^	**<0.01**	7.07 × 10^–4^ ± 1.42 × 10^–3^	>0.1
*T*_1_	23.97 ± 1.69	**<0.01**	0.45 ± 0.10	**<0.01**	−17.30 ± 71.21	>0.1
*T*_2_	2.13 ± 0.16	**<0.01**	1.44 × 10^–2^ ± 8.83 × 10^–3^	>0.1	−2.87 ± 6.68	>0.1
FA	−1.18 × 10^–3^ ± 1.19 × 10^–4^	**<0.01**	−2.45 × 10^–5^ ± 6.42 × 10^–6^	**<0.01**	−4.14 × 10^–3^ ± 4.88 × 10^–3^	>0.1
MD	1.10 × 10^–5^ ± 8.58 × 10^–7^	**<0.01**	1.86 × 10^–7^ ± 4.64 × 10^–8^	**<0.01**	9.15 × 10^–5^ ± 3.53 × 10^–5^	**<0.05**
CBF	−0.27 ± 0.05	**<0.01**	−6.77 × 10^–3^ ± 2.86 × 10^–3^	**<0.05**	−3.63 ± 2.01	**<0.1**

*Bold indicates *p* < 0.05.*

**FIGURE 3:**
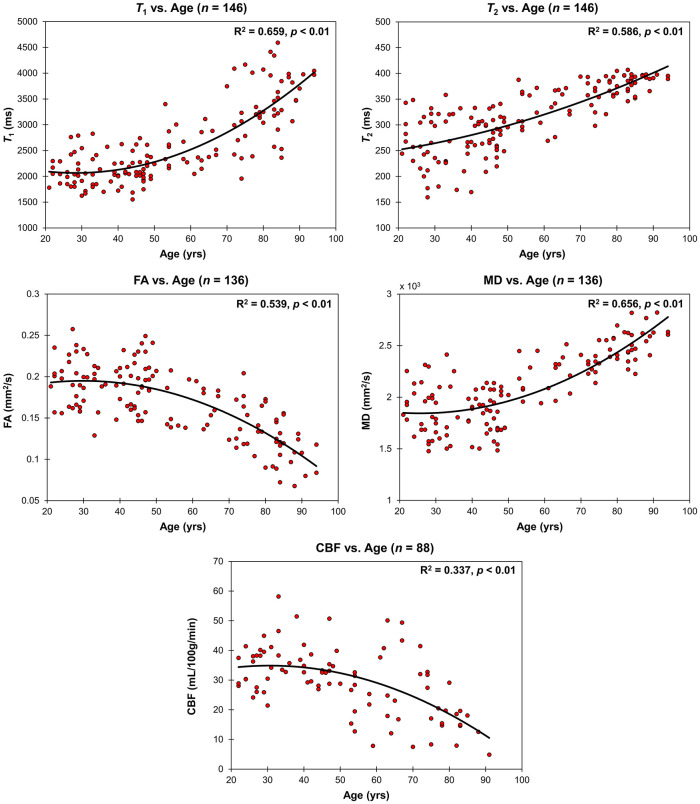
Regressions of relaxometry measures (T_1_ and T_2_), DTI measures (FA and MD), or CBF with age. For each plot, the coefficient of determination, *R*^2^, and *p*-value, *p*, are reported. T_1_, T_2_ and MD exhibit non-linearly increasing trends with age while FA and CBF exhibit a non-linearly decreasing trends with age.

Figure 3 shows the relaxometry measures, *T*_1_ and *T*_2_ (*n* = 146), the DTI measures, FA and MD (*n* = 136), and CBF (*n* = 88) for all participants within the CP as a function of age. Multiple linear regression analysis showed that the effect of age was statistically significant on all of these MRI measures (Table 1). Furthermore, besides *T*_2_, the quadratic effect of age on all MR measures was statistically significant, with FA (*p* < 0.01) and CBF (*p* < 0.05) exhibiting non-linearly decreasing trends with age, while *T*_1_ (*p* < 0.01) and MD (*p* < 0.01) exhibiting non-linearly increasing trends with age (Table 1 and Figure 3). Finally, sex effect on MD and CBF was statistically significant, with women exhibiting higher CBF values and lower MD values as compared to men.

**FIGURE 4:**
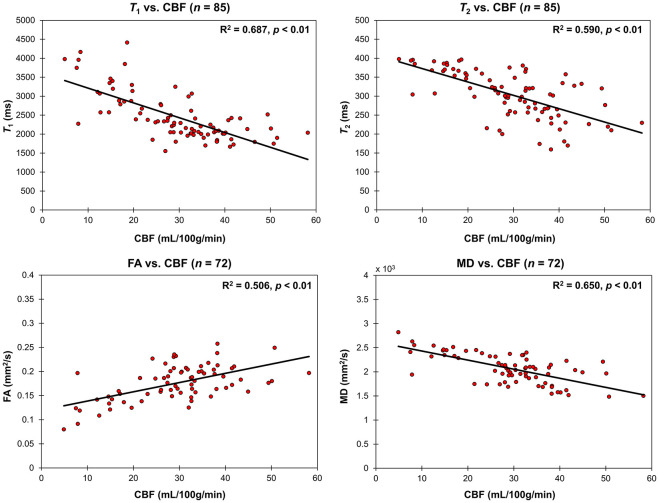
Regressions of relaxometry measures (T_1_ and T_2_) or DTI measures (FA and MD) with CBF in the CP. For each plot, the coefficient of determination, *R*^2^, and *p*-value, *p*, are reported. T_1_, T_2_, and MD all linearly decreased with CBF while FA linearly increased with CBF.

Correlations between relaxometry or DTI measures and CBF were assessed using multiple linear regression analyses while accounting for the effect of age. As shown in Figure 4 and Table 2, CBF was significantly correlated with FA exhibiting a linearly increasing trend with increasing CBF, while *T*_1_, *T*_2_, and MD exhibiting linearly decreasing trends with increasing CBF (Table 2 and Figure 4).

**TABLE 2:** Coefficients of regression ± standard error (SE) and *p*-values of age, age^2^, and CBF of the multiple linear regression analysis of T_1_, T_2_, FA, or MD.

	Multiple linear regression model parameters					
	β _*age*_ ± SE_*age*_	*p*_*age*_	β _*age*_^2^ ± SE_*age*_^2^	*p*_*age*_^2^	β _*CBF*_ ± SE_*CBF*_	*p*_*CBF*_
*T*_1_	14.954 ± 2.32	**<0.01**	0.43 ± 0.12	**<0.01**	−19.96 ± 4.53	**<0.01**
*T*_2_	1.66 ± 0.26	**<0.01**	1.27 × 10^–3^ ± 1.32 × 10^–3^	>0.1	−1.83 ± 0.50	**<0.01**
FA	−8.53 × 10^–4^ ± 2.07 × 10^–4^	**<0.01**	−2.06 × 10^–5^ ± 1.09 × 10^–5^	**<0.1**	1.22 × 10^–3^ ± 4.13 × 10^–4^	**<0.01**
MD	8.59 × 10^–6^ ± 1.40 × 10^–6^	**<0.01**	9.28 × 10^–8^ ± 7.36 × 10^–8^	>0.1	−1.41 × 10^–5^ ± 2.80 × 10^–6^	**<0.01**

*Bold indicates *p* < 0.05.*

## Discussion

In this cross-sectional study, we assessed sex and age-related microstructural and functional differences that occur in the CP using a combination of robust MRI measures in a large cohort of cognitively unimpaired subjects (*n* = 155) spanning 21–94 years of age. We found significant positive association between CP volume and age. We attribute this novel observation to the functional and morphological alterations that occur within the CP as well as to ventriculomegaly and, perhaps, inflamm“aging,” which are common features of normal brain aging. Structurally, the CP is known to undergo numerous changes including accumulation of calcifications and lipofuscin deposits as well as thickening of the stroma (Shuangshoti and Netsky, 1970; Serot et al., 2001; Yalcin et al., 2016). Functionally, CSF production and turnover decrease with age in the CP (Preston, 2001; Stoquart-ElSankari et al., 2007). Thus, an increase in CP volume may serve as a compensatory or protective mechanism to morphological disruptions and the diminished ability to produce CSF (Redzic et al., 2005). The CP also plays a critical role in producing growth factors (e.g., insulin, IGF1, and IGF2) necessary for normal brain development and regulating its own growth and maintenance of epithelial cell health. While it has been observed that epithelial cell growth of the CP slows with age in younger animals (McDonald and Green, 1988; Liddelow et al., 2010), previous reports have demonstrated that enlarged ventricles and increasing inflammation due to stroke or direct tissue injury upregulated CP-derived growth factors resulting in volume proliferation of CP epithelial cells (Li et al., 2002; Barkho and Monuki, 2015). Further, previous studies using similar segmentation methods have found a positive association between increasing LV size and CP volume when comparing healthy controls to a disease state (Egorova et al., 2019; Lizano et al., 2019; Tadayon et al., 2020). Due to the multifunctional role of the CP, it is difficult to discern the dominant source and purpose of volume enlargement during normative aging, which necessitates further investigations.

Our finding of higher MD values with age is consistent with Alicioglu et al. (2017). This age-related increase in MD is further supported by our findings that FA significantly decreased with age and that *T*_1_ and *T*_2_ significantly increased with age. MD describes the overall diffusion and motion of water molecules in the brain, with higher MD values reflect more water mobility (Alexander et al., 2007; Salat, 2014). Similarly, *T*_1_ and *T*_2_ both depend on water content with higher values are also associated with more water mobility (Deoni, 2010). FA, on the other hand, is used to describe anisotropy with low values associated with less restricted movement of molecules (Alexander et al., 2007; Salat, 2014), with lower values of FA with age here are likely due to microstructural alterations or damage to the CP epithelium, leading to increased isotropy of water diffusion. Taken together, we provisionally attribute these observed changes in relaxometry and DTI measures primarily to structural alterations to the CP microstructure and, potentially, to a decreased structural integrity of the BCSFB with advanced age. Indeed, it has been observed in animals that the BCSFB becomes leakier with increasing age, specifically for compounds that were low and medium in molecular weight (Chen et al., 2009). Possible sources for structural alterations in the BCSFB stem from morphological disruptions, as previously described, as well as increasing inflammation. Indeed, previous work showed that aging was associated with a shift toward the Th2 response, which consequently resulted in compromised epithelial tight junctions that are vital in maintaining the structural integrity of the BCSFB (Baruch et al., 2013). However, additional investigations, especially dynamic contrast-enhanced-based measures as well as histological studies, are required to support our conjecture of a potential association between decreased structural integrity of CP and higher BCSFB permeability.

Interestingly, our MD measure exhibited significant differences between men and women, with men exhibiting higher MD values. Alicioglu et al. (2017) observed that women experience a steeper increase in MD after the age of 61 as compared to men. Further, several studies have shown that women exhibit higher CBF as compared to men (Devous et al., 1986; Rodriguez et al., 1988; Podreka et al., 1989; Gur and Gur, 1990; Esposito et al., 1996; Parkes et al., 2004; Liu et al., 2012, 2016; Alisch et al., 2021). Sex differences in CP microstructure and function would be expected. Indeed, in addition to reduced CSF turnover, it is suggested that a dysfunctional BCSFB contributes to greater protein content in the CSF during aging (Chen et al., 2009). In fact, previous reports found that men had a significantly higher CSF/serum albumin ratio (QAlb), a biomarker of BCSFB permeability, compared to women which demonstrates a greater loss of integrity in men (Parrado-Fernandez et al., 2018; Castellazzi et al., 2020). The current scientific literature on sex differences within the CP remain limited, but we speculate that sex hormones may play a role in BCSFB permeability (Santos et al., 2017). Further investigations are required to elucidate sex-related differences in CP’s microstructure and function.

We also observed an age-related decrease in CBF in the CP, likely reflecting changes in CP morphology or in the hormonal and neurotransmitter levels. CP blood flow is regulated by several molecules including arginine vasopressin, angiotensin II, dopamine, and serotonin. Arginine vasopressin and angiotensin II have an inhibitory effect on blood in the CP (Faraci et al., 1988; Maktabi et al., 1990), while dopamine and serotonin are both known to increase blood flow (Townsend et al., 1984; Faraci et al., 1989). Previous studies have showed that aging is associated with increases in arginine vasopressin (Frolkis et al., 2000) and that angiotensin II induces the release of vasopressin (Keil et al., 1975; Qadri et al., 1993), thus indicating a potential mechanism for the age-related decline in CP blood flow. Further, dysfunctional binding and transport of dopamine and serotonin, as observed in other parts of the brain (Meltzer et al., 1998; Karrer et al., 2017), may contribute to decreased CBF. Moreover, morphological examinations show that the arterial walls are thickened and that there is a reduction in blood vessel density during normal aging which leads to limited contact between the blood and the CP epithelial cell layer (Van Cauwenberghe et al., 2020). Finally, our results indicate that CBF was associated with both relaxometry and DTI measures, suggesting that low CBF is associated with the reduced structural integrity of the CP. Indeed, adequate CBF is paramount for nutrient and oxygen delivery and clearance of metabolic by-products; dysfunction of this ability is a characteristic feature of neurodegenerative diseases.

Although we examined a relatively large cohort and used advanced MR methodology, our study has limitations. Our dataset is cross-sectional so that the observed trends in the CP microstructure and function with age as well as the associations between CBF and the other MRI measures requires further validation through longitudinal studies. Such work, motivated by the present results, is underway. Furthermore, our analysis of functional and structural differences in CP with aging were limited to the LVs. We note that CSF partial volume effects may bias derived parameter values. More accurate automated segmentation methods, including the third and fourth ventricles, as well as higher resolution structural images are needed for a better evaluation. In addition, our DTI-related results must be interpreted with caution. Indeed, given the large fraction of free water in the CP, this could have introduced bias in derived parameter values. Although the free-water elimination DTI (FWE-DTI) approach has been used widely to distinguish free-water partial-volume effects from tissue’s diffusion in healthy aging and degenerative diseases (Pasternak et al., 2009), it has recently been shown that this method is unstable when applied to single-shell DTI data, requiring careful implementation (Golub et al., 2021). Further, we note that our derived DTI parameters values could change depending on the choice of the *b*-values. Several DTI protocols involve use of *b*-values of 0 and 1000 s/mm^2^ while others incorporate lower *b*-values. Our choice of using lower *b*-values was driven by the desire to minimize non-Gaussian diffusion. Moreover, we used identical ASL experimental parameters for all subjects and acquired CBF images at a single post-labeling delay, implicitly assuming negligible effects of spatial variation in arterial transit time (ATT), the time of the arterial bolus to transit from the labeling plane to the imaging volume (Duvernoy et al., 1981; Qin et al., 2014). Although this is a reasonable assumption (Alsop et al., 2015), ATT may vary spatially and may differ between subjects due to arterial blood velocity differences; this could introduce a small bias in derived CBF values. A multiple post-labeling delay ASL technique may provide more accurate CBF determination (Qin et al., 2014). Finally, other factors such as inflammatory markers, blood pressure, and medications were not considered in this work.

## Conclusion

We examined age-related differences in CP microstructure and function in a cohort of cognitively unimpaired participants across a wide age range using quantitative MRI. We showed that aging was associated with increases in volume and decreases in blood flow or structural integrity. This work lay the foundation for further investigation of the functional and structural changes of the CP with normal aging and neuropathology.

## Data Availability Statement

The raw data supporting the conclusions of this article will be made available by the authors, without undue reservation.

## Ethics Statement

This study including our MRI protocol was approved by the MedStar Research Institute and the National Institutes of Health Intramural Ethics Committees, and all examinations were performed in compliance with the standards established by the National Institutes of Health Institutional Review Board. The patients/participants provided their written informed consent to participate in this study.

## Author Contributions

JE and MB: research design, results interpretation, and manuscript writing and editing. JA, JE, MK, CT, MA, ZG, and NK: analysis, results interpretation, and manuscript writing and editing. All authors contributed to the article and approved the submitted version.

## Conflict of Interest

The authors declare that the research was conducted in the absence of any commercial or financial relationships that could be construed as a potential conflict of interest.

## Publisher’s Note

All claims expressed in this article are solely those of the authors and do not necessarily represent those of their affiliated organizations, or those of the publisher, the editors and the reviewers. Any product that may be evaluated in this article, or claim that may be made by its manufacturer, is not guaranteed or endorsed by the publisher.

## Footnotes

1
http://surfer.nmr.mgh.harvard.edu/
